# VHL-1 inactivation and mitochondrial antioxidants rescue *C. elegans* dopaminergic neurodegeneration

**DOI:** 10.1007/s13238-019-0621-4

**Published:** 2019-04-03

**Authors:** Song Chen, Shuo Luo, Zhe Zhang, Dengke K. Ma

**Affiliations:** 1 Cardiovascular Research Institute and Department of Physiology, University of California San Francisco (UCSF), San Francisco, CA 94158 USA; 2 Jiangsu Key Laboratory of Druggability of Biopharmaceuticals, State Key Laboratory of Natural Medicines, School of Life Science and Technology, China Pharmaceutical University, Nanjing, 210009 China

**Dear Editor,**

Mitochondrial complex I is important for cellular ATP production by transporting electrons and generating proton gradient across the mitochondrial inner membrane (Hirst, 2013). It is also a major cellular locus where electron leakage to oxygen produces superoxide, an ROS (reactive oxygen species), particularly under oxidative stress conditions. Dysfunctional complex I contributes to the most common oxidative phosphorylation disorder in humans, with many identified genetic mutations in complex I subunits causing a variety of human disorders including Leigh syndrome, encephalomyopathy, cardiomyopathy, parkinsonism and hereditary optic neuropathy (Hirst, 2013; Guo et al., 2017). In addition, complex I is the major target of many parkinsonism-causing neurotoxins including rotenone and MPTP. Past biochemical, cell biological and structural studies have elucidated how complex I functions normally in mitochondrial respiration (Hirst, 2013; Guo et al., 2017; Letts and Sazanov, 2017). Nonetheless, our understanding of mechanisms how complex I dysfunction leads to human diseases is far from completion; therapeutic targets and strategies are urgently needed.

Here, we use the genetically tractable *C*. *elegans* to develop a novel disease model for mitochondrial complex I dysfunction-induced dopaminergic neurodegeneration. We found that partial loss-of-function of a key complex I subunit NDUF-7 caused dopaminergic neurodegeneration in a dopamine neuron subtype-specific manner. In exploring “therapeutic” strategies to treat such “Parkinsonism” model, we show that genetic deletion of the *C*. *elegans* orthologue of VHL (von Hippel-Lindau) or pharmacologically providing mitochondria-targeting antioxidants can effectively rescue dopaminergic neurodegeneration. Our findings reveal neuron subtype-specific dopaminergic neurodegeneration caused by mitochondrial complex I dysfunction and identify novel protein targets and promising pharmacological leads to treat complex I dysfunction-related mitochondrial disorders.

To examine phenotypic consequences of complex I dysfunction on dopamine neurons, we generated a compound *C*. *elegans* strain with two integrated genetic markers of fluorescent proteins, including *zcIs9* [*hsp-60p*::*GFP*] to monitor the mitochondrial unfolded protein response (mito-UPR), *otIs181* to mark all 8 dopamine neurons (4 CEP, 2 ADE and 2 weakly fluorescent PDE neurons) and 2 non-dopaminergic AIY neurons (Fig. 1A and 1B), and with a partial loss-of-function mutation of the gene *nduf-7* (Flames and Hobert, 2009; Rauthan et al., 2015). *nduf-7* encodes a protein orthologous to NDUFS7, the NADH Ubiquinone oxidoreductase Fe-S protein, one of the 45 highly conserved protein subunits of mitochondrial complex I. *nduf-7*(*et19*) is a nonsense mutation that removes the last 5 highly conserved amino acids of NDUF-7 and causes reduced mitochondrial complex I activity and constitutive activation of mito-UPR (Rauthan et al., 2015).

**Figure 1:**
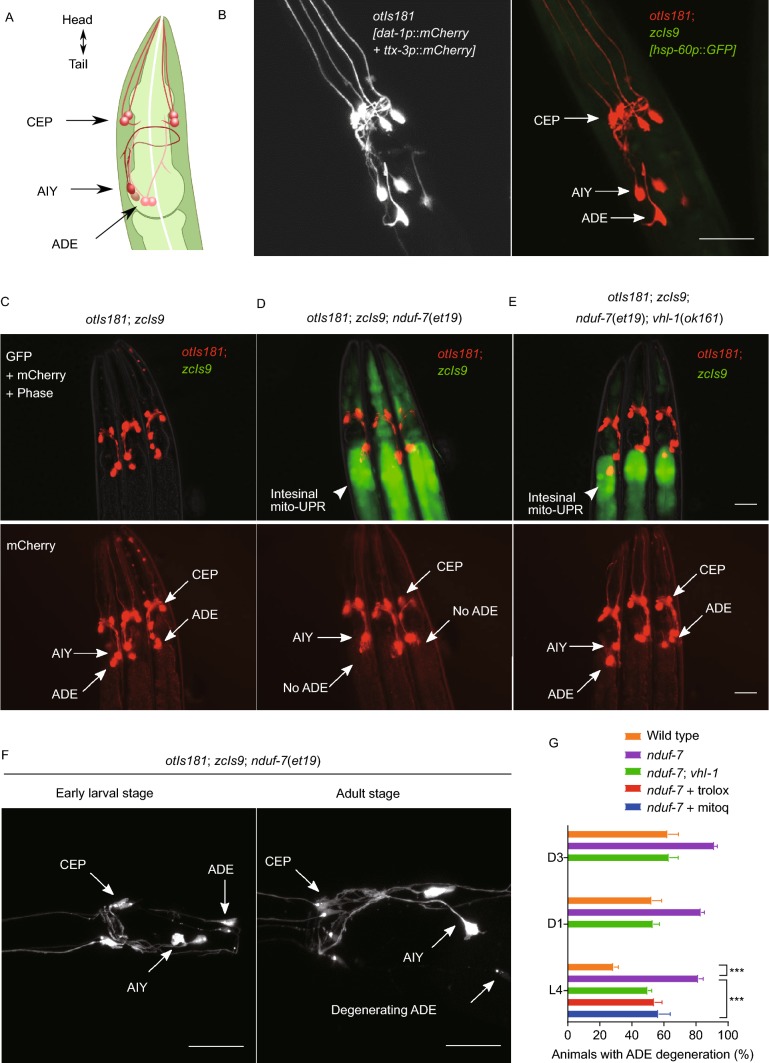
**Genetic and pharmacological rescue of complex I-dysfunction induced ADE neurodegeneration**. (A) Schematic illustrating the four CEP, two ADE dopaminergic and two non-dopaminergic AIY neurons. (B) Exemplar confocal fluorescence images of L4-stage transgenic strains with the markers *otIs181* and *zcIs9* to monitor dopamine neuron morphologies and mito-UPR, respectively, in the wild-type genetic background. (C) Merged GFP, mCherry and phase-contrast (up) and mCherry only (down) epifluorescence whole-body images showing intact CEP, ADE and AIY neurons, marked by mCherry, and absence of mito-UPR, marked by GFP in L4-stage wild type animals. (D) Exemplar images taken under the same condition as in (C) showing intact CEP and AIY, loss of ADE neurons and constitutive mito-UPR in L4-stage *nduf-7*(*et19*) mutants. (E) Exemplar images taken under the same condition as in (C) showing intact CEP and AIY, rescued ADE neurons and constitutive mito-UPR in L4-stage *nduf-7*(*et19*); *vhl-1*(*ok161*) double mutants. (F) Exemplar confocal images showing progressive age-dependent ADE neurodegeneration. (G) Quantification of ADE neurodegeneration in various strains of indicated genotype and with pharmacological treatments. Vehicle-only control is similar to that without pharmacological treatments. Both mitoQ and trolox were applied to the culture plates (0.3 and 3 mg/mL, respectively) with exposure to animals at 20 °C before scoring ADE neurons. ***P < 0.001, ANOVA; *n* ≥ 3 biological replicates with animal numbers of each group ≥30. Scale bars, 50 µm

We first confirmed that *nduf-7*(*et19*) caused constitutive mito-UPR in *C*. *elegans* with strong *zcIs9* [*hsp-60p*::*GFP*] expression in the intestine (Fig. 1C and 1D). To examine potential dopamine neuron phenotypes of *nduf-7*(*et19*) mutants, we characterized the mCherry-marked cells in both larval L4 and adult stages of animals and compared the *nduf-7* mutant with wild type. *nduf-7*(*et19*) did not appear to affect the 4 CEP neurons or the 2 AIY neurons, based on their morphologies and characteristic locations in the animal at the stage L4 (Fig. 1D). By contrast, we found that the two ADE neurons from L4-stage *nduf-7*(*et19*) mutants were strikingly missing in a large fraction of animals (exemplar images shown in Fig. 1D). The larval L3-stage *nduf-7*(*et19*) mutants also exhibited ADE neuron loss but to a lesser degree. Two PDE neurons have very weak mCherry signal, even in wild type animals. CEP neurons became loss at a much later stage (i.e., day 3 adult). AIY neurons were morphologically normal throughout development in WT and mutant animals. Thus, for the remainder of the study, we focused on characterizing how the marked ADE neuron loss (or neurodegeneration, see below) in *nduf-7*(*et19*) mutants can be rescued by genetic and pharmacological means.

In humans, mutations in NDUFS7 cause Leigh syndrome, a severe neurological disorder that is characterized by bilaterally symmetrical necrosis and neurodegeneration in subcortical brain regions. Recent studies showed that chronic hypoxia exposure can be an effective therapy to treat mouse models of Leigh syndrome (Ferrari et al., 2017; Jain et al., 2016). To test directly whether HIF (hypoxia inducible factor, the master transcriptional mediator of cellular hypoxic response) can modify ADE neurodegeneration, we generated another compound *C*. *elegans* strain with *zcIs9*; *otIs181*; *nduf-7*(*et19*); *vhl-1*(*ok161*). *ok161* is a genetic deletion that causes loss-of-function of VHL, the sole E3 ubiquitin ligase to degrade HIF, and leads to HIF stabilization and constitutive activation of HIF target genes (Shen and Powell-Coffman, 2003). We found that *vhl-1*(*ok161*) markedly suppressed effects of *nduf-7*(*et19*) in causing the ADE neuron loss (Fig. 1E). CEP, AIY and ADE neurons in larval L2-stage *nduf-7*(*et19*) mutants were largely normal compared with those in the adult stage (Fig. 1F), supporting that the lost ADE neurons were indeed caused by neurodegeneration, rather than defective dopamine neuron specification and differentiation. ADE neurodegeneration appears to be progressive over time in wild type and worsened in *nduf-7*(*et19*) mutants and restored in *nduf-7*(*et19*); *vhl-1*(*ok161*) double mutants (Fig. 1G). These results show that VHL-1 inactivation by genetic means can rescue ADE neurodegeneration caused by *nduf-7* mutations.

Next we sought pharmacological agents that can rescue the *nduf-7*(*et19*)-caused ADE neurodegeneration. We treated the *zcIs9*; *otIs181*; *nduf-7*(*et19*) animals with a panel of 14 diverse compounds of mitochondrial modulators and identified two compounds that were effective in rescue: mitoQ and trolox (Murphy and Smith, 2007). MitoQ is a ubiquinone derivative that accumulates in mitochondria owing to the covalent attachment of the cation triphenylphosphonium. Trolox is a cell membrane-permeable, water-soluble derivative of vitamin E with potent antioxidant properties. We cultivated *zcIs9*; *otIs181*; *nduf-7*(*et19*) animals with exposure to these two compounds for 24 h prior to scoring ADE neurons at L4 stage and found that both treatments effectively alleviated ADE neurodegeneration (Fig. 1G). Such effects were particularly pronounced in L4-stage animals, indicating an optimal time window of treatment before neurodegeneration becomes irreversible. These results show that mitochondrial antioxidants when administered early enough can alleviate ADE neurodegeneration, which is caused by *nduf-7* mutations likely through impaired mitochondrial respiratory functions.

Loss of *vhl-1* strongly rescued ADE neurodegeneration but not the constitutive mito-UPR of *nduf-7* mutants (Fig. 1E), suggesting that mechanism of rescue by loss of *vhl-1* might be independent of mito-UPR and/or ROS. The superoxide anion, a major type of ROS, is generated as a by-product of mitochondrial oxidative phosphorylation and its levels can indicate mitochondrial states of respiratory functions. We used a fluorescent dye MitoSOX red to measure steady-level superoxide levels in *nduf-7* and *nduf-7*; *vhl-1* mutants. We found that the posterior bulbs of *nduf-7* mutants exhibited lower MitoSOX signals than wild type (Fig. 2A), indicating that mitochondrial respiratory activity is decreased, consistent with an important role of NDUF-7 for mitochondrial complex I functions. Reduced MitoSOX signals in *nduf-7* mutants also suggest that mitochondrial antioxidants rescue ADE neurodegeneration likely through restored mitochondrial respiratory function rather than alleviating excessive superoxide toxicity. Furthermore, we found that *nduf-7*; *vhl-1* mutants exhibited higher MitoSOX signals than *nduf-7* single mutants (Fig. 2A and 2B), indicating restored mitochondrial respiratory function. Taken together, these results suggest that mechanisms of rescue of ADE neurodegeneration by *vhl-1* inactivation likely act through modulating mitochondrial respiratory functions.

**Figure 2:**
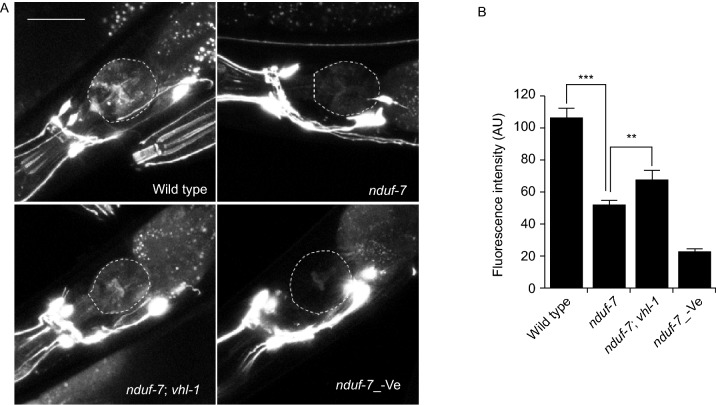
**VHL-1 inactivation rescues impaired mitochondrial respiratory activity in** ***nduf-7*** **mutants**. (A) Exemplar confocal images showing MitoSOX Red staining of posterior pharyngeal bulbs (circled dash line). The CEP, ADE and AIY neurons are labeled by *dat-1p*::*mCherry* reporter transgenes and are also red fluorescent, precluding direct MitoSOX measurements in neurons. For each genotype (Wild type, *nduf-7*, *nduf-7*; *vhl-1*, and *nduf-7*_-Ve, with vehicle only control), twenty L4-stage animals were transferred to MitoSOX Red plates seeded with 100 mL of OP50 *E*. *coli* supplemented with 50 μmol/L MitoSOX Red (M36008, ThermoFisher Scientific) and incubated for 24 h at 20 °C in darkness. Confocal images of animal’s posterior pharyngeal bulb were processed in ImageJ and fluorescence intensity of maximal z-projection of posterior pharyngeal bulbs was measured individually. Scale bars: 25 µm. (B) Quantification of MitoSOX Red staining of posterior pharyngeal bulbs in various strains of indicated genotype and conditions. Bars represent mean ± S.E.M. **P < 0.01; ***P < 0.001, ANOVA

Although numerous lines of evidence support causal roles of mitochondrial complex I dysfunction in the pathogenesis of Parkinson’s diseases, deletion of *NDUFS4*, a key complex I component, globally or selectively in dopamine neurons did not result in apparent overall dopamine neuron loss in mice (Choi et al., 2017). In *C*. *elegans*, we found that complex I dysfunction by genetic mutation in *nduf-7* causes degeneration of ADE neurons but not the other 4 CEP dopamine neurons at early stages of animals, although we did observe that CEP neurons also degenerate at later stages. Our findings suggest that complex I dysfunction might also cause dopamine neuron subtype-specific progression of degeneration at different ages and to different degrees in mammals. We further identify *vhl-1* as a genetic modifier and mitochondria-targeting antioxidants as rescuing pharmacological agents for this previously undescribed dopamine neuron subtype-specific neurodegeneration in *C*. *elegans*. Whether rescue by VHL inactivation and mitochondrial antioxidants can be recapitulated by hypoxia treatment or HIF activation remain to be investigated. Based on our findings, we propose that VHL inactivation and mitochondrial antioxidants protect against complex I dysfunction-induced neurodegeneration by modulating mitochondrial respiratory activity. Therapeutically, inhibitors of VHL and mitochondrial antioxidants may lead to development of potential treatments for Parkinson’s diseases and more broadly complex I dysfunction-related mitochondrial disorders.

## References

[CR1] Choi W-S, Kim H-W, Tronche F, Palmiter RD, Storm DR, Xia Z (2017). Conditional deletion of Ndufs4 in dopaminergic neurons promotes Parkinson’s disease-like non-motor symptoms without loss of dopamine neurons. Sci Rep.

[CR2] Ferrari M, Jain IH, Goldberger O, Rezoagli E, Thoonen R, Cheng KH, Sosnovik DE, Scherrer-Crosbie M, Mootha VK, Zapol WM (2017). Hypoxia treatment reverses neurodegenerative disease in a mouse model of Leigh syndrome. Proc Natl Acad Sci USA.

[CR3] Flames N, Hobert O (2009). Gene regulatory logic of dopaminergic neuron differentiation. Nature.

[CR4] Guo R, Zong S, Wu M, Gu J, Yang M (2017). Architecture of human mitochondrial respiratory megacomplex I2III2IV2. Cell.

[CR5] Hirst J (2013). Mitochondrial complex I. Annu Rev Biochem.

[CR6] Jain IH, Zazzeron L, Goli R, Alexa K, Schatzman-Bone S, Dhillon H, Goldberger O, Peng J, Shalem O, Sanjana NE (2016). Hypoxia as a therapy for mitochondrial disease. Science.

[CR7] Letts JA, Sazanov LA (2017). Clarifying the supercomplex: the higher-order organization of the mitochondrial electron transport chain. Nat Struct Mol Biol.

[CR8] Murphy MP, Smith RAJ (2007). Targeting antioxidants to mitochondria by conjugation to lipophilic cations. Annu Rev Pharmacol Toxicol.

[CR9] Rauthan M, Ranji P, Abukar R, Pilon M (2015). A mutation in *Caenorhabditis elegans NDUF-7* activates the mitochondrial stress response and prolongs lifespan via ROS and CED-4. G3 Genes Genomes Genet.

[CR10] Shen C, Powell-Coffman JA (2003). Genetic analysis of hypoxia signaling and response in *C. elegans*. Ann N Y Acad Sci.

